# Clinical Aspects, Imaging Features, and Considerations on Bisphosphonate-Related Osteonecrosis Risk in a Pediatric Patient with Osteogenesis Imperfecta

**DOI:** 10.1155/2014/384292

**Published:** 2014-08-26

**Authors:** Fábio Wildson Gurgel Costa, Filipe Nobre Chaves, Alexandre Simões Nogueira, Francisco Samuel Rodrigues Carvalho, Karuza Maria Alves Pereira, Lúcio Mitsuo Kurita, Rodrigo Rodrigues Rodrigues, Cristiane Sá Roriz Fonteles

**Affiliations:** ^1^Division of Oral Radiology, Department of Clinical Dentistry, Federal University of Ceará, Rua Alexandre Baraúna 949, 60430-160 Fortaleza, CE, Brazil; ^2^Division of Stomatology and Oral Radiology, Department of Clinical Dentistry, Federal University of Ceará, Rua Alexandre Baraúna 949, 60430-160 Fortaleza, CE, Brazil; ^3^Division of Stomatology, Department of Clinical Dentistry, Federal University of Ceará, Rua Alexandre Baraúna 949, 60430-160 Fortaleza, CE, Brazil; ^4^Division of Oral and Maxillofacial Surgery, Department of Oral and Maxillofacial Surgery, Walter Cantídio University Hospital, Federal University of Ceará, Rua Alexandre Baraúna 949, 60430-160 Fortaleza, CE, Brazil; ^5^Division of Oral Pathology and Stomatology, Department of Clinical Dentistry, Federal University of Ceará, Rua Alexandre Baraúna 949, 60430-160 Fortaleza, CE, Brazil; ^6^Division of Oral and Maxillofacial Surgery, Department of Oral and Maxillofacial Surgery, Federal University of Rio Grande do Norte, Campus Universitário Lagoa Nova, 59078-900 Natal, RN, Brazil; ^7^Division of Pediatric Dentistry, Department of Clinical Dentistry, Federal University of Ceará, Rua Alexandre Baraúna 949, 60430-160 Fortaleza, CE, Brazil

## Abstract

Osteogenesis imperfecta (OI) is a rare hereditary condition caused by changes in collagen metabolism. It is classified into four types according to clinical, genetic, and radiological criteria. Clinically, bone fragility, short stature, blue sclerae, and locomotion difficulties may be observed in this disease. OI is often associated to severe dental problems, such as dentinogenesis imperfecta (DI) and malocclusions. Radiographically, affected teeth may have crowns with bulbous appearance, accentuated constriction in the cementoenamel junction, narrowed roots, large root canals due to defective dentin formation, and taurodontism (enlarged pulp chambers). There is no definitive cure, but bisphosphonate therapy is reported to improve bone quality; however, there is a potential risk of bisphosphonate-related osteonecrosis of the jaw. In this study we report a case of OI in a male pediatric patient with no family history of OI who was receiving ongoing treatment with intravenous perfusion of bisphosphonate and who required dental surgery. In addition, we discussed the clinical and imaging findings and briefly reviewed the literature.

## 1. Introduction

Osteogenesis imperfecta (OI) is a rare hereditary autosomal dominant disorder caused by mutations in the collagen type I alpha1 (COL1A1) or collagen type I alpha2 (COL1A2) genes associated with type I collagen metabolism changes. It is reported with an incidence of 1 : 20,000 to 1 : 40,000 births [1, 2]. Craniofacial and dental disorders may include growth deficiency, ligamentous laxity, blue sclerae, hearing loss, dentinogenesis imperfecta (DI), or a combination of these features [2]. There is no definitive cure for OI, but bisphosphonate therapy is reported to improve mobility and bone density and to reduce pain and the incidence of fracture [3, 4]. These drugs are nonmetabolized analogs of pyrophosphate related to bone, used to prevent or ameliorate skeletal complications in serious diseases such as osteoporosis, bone Paget's disease, hypercalcemia related to malignancies, bone metastases, and multiple myeloma [5].

In addition, bisphosphonates are used in children and adolescents with OI [6]. However, their use may also favor the development of osteonecrosis of the jaw, especially following dental surgery [5, 6]. In this study we report a case of OI in a male pediatric patient with no family history of OI who was receiving ongoing treatment with intravenous perfusion of bisphosphonate and who required dental surgery.

## 2. Case Report

**Figure 1:**
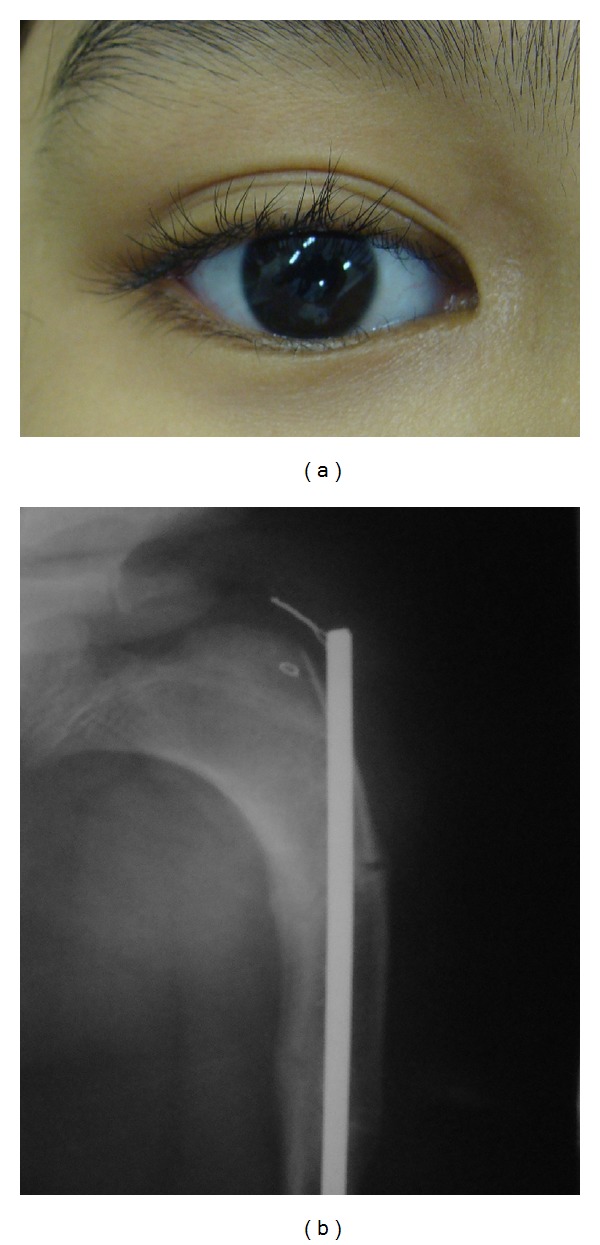
Detail of the blue sclera, a common clinical finding in type IV osteogenesis imperfecta (a), and deformed limbs with arched appearance with medical history of bone fractures (b).

**Figure 2:**
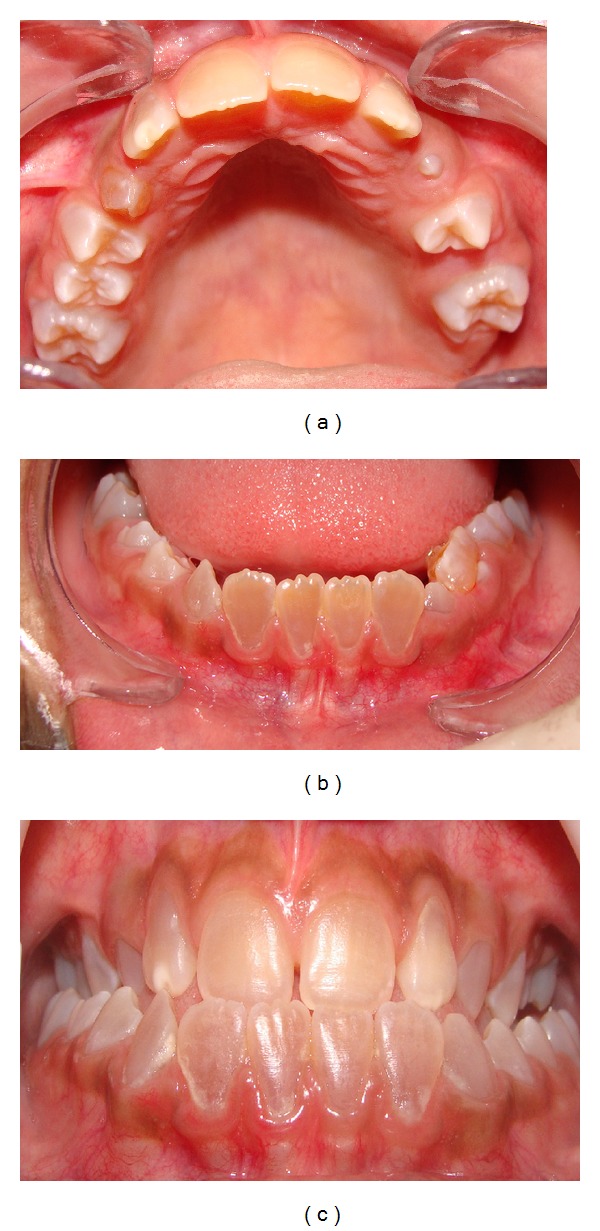
Anterior teeth showing brownish discoloring, a common clinical finding in type I dentinogenesis imperfecta. Upper dental arch (a), lower arch (b), and dental occlusion (c).

**Figure 3:**
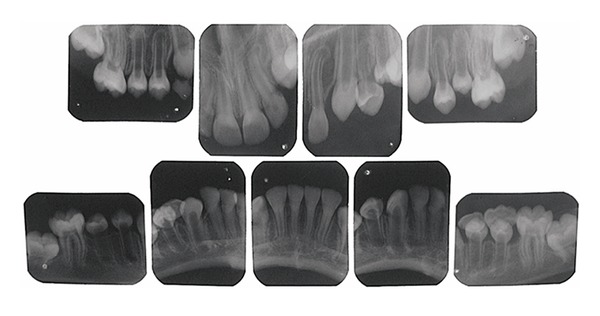
Periapical X-ray showing taurodontic teeth with enlarged pulp chambers and insidious early obliteration of the coronary pulp chambers of the lower incisors.

**Figure 4:**
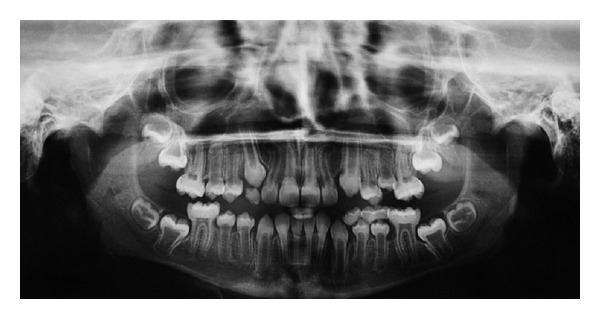
Panoramic radiography showing large pulp chambers in all teeth, and absence of agenesis, impaction, or supernumerary roots.

A 10-year old boy with OI presented at the Stomatology service of the Federal University of Ceará (Sobral). The OI type was not identified, the genetic test was inconclusive, and the patient had no family history of OI. Physical findings included short stature, blue sclerae, and locomotion difficulties due to deformed and arched limbs (Figure 1). The medical history included reports of 16 bone fractures and intravenous administration of pamidronate disodium infused over a period of 3 days and repeated every 4 months. Anterior and posterior cross-bites, Angle class III malocclusion, and mild mandibular prognathism were observed on oroscopy. The anterior teeth were brownish, especially the lower incisors. Hypomineralization was observed on the vestibular surface of the upper left lateral incisor and a gray band was seen on the posterior teeth, especially the molars, suggesting DI (Figure 2). The remaining deciduous teeth (the upper right maxillary canine and the right mandibular molars) were more brownish than the permanent teeth and were undergoing exfoliation, requiring extraction. Periapical radiography revealed enlarged pulp chambers, with insidious early obliteration of the coronary pulp chambers of the lower incisors and a thin layer of dentin deposited along all the teeth producing a taurodontic appearance. The teeth had ample crowns and cervical constrictions, with molars taking on a bell-like shape resembling a crown (Figure 3). Panoramic radiography confirmed large pulp chambers in all teeth, but no agenesis, impaction, or supernumerary roots (Figure 4). The primary mandibular molar teeth (first and second left mandibular teeth) presented physiologic mobility for some time, but were retained in spite of the underlying erupting premolars, which generated dental plaque accumulation, pain, and gingival bleeding during mastication and tooth brushing. Thus, these teeth were extracted to prevent further discomfort and to allow proper dental eruption. The child's mother requested the extraction of the primary maxillary right canine, in fear of having her son present the previously described findings. Surgery was performed without suspending bisphosphonate therapy and without antibacterial prophylaxis since no risk of infection was perceived in the transsurgical period. Currently, after 18 months of follow-up, the patient shows no signs of osteonecrosis.

## 3. Discussion

OI is a hereditary connective tissue disorder, also known as “brittle bone disease”, and it is classified according to the clinical, genetic, and radiological criteria (Table 1) [1, 2]. Children usually display clinical and imaging changes in the dentition, commonly DI features (gray-brown friable teeth, bulky crowns, and early calcification of the pulpal space), opalescent and discolored teeth (ranging from brownish-yellow to gray), and malocclusion (drastic open bites and impacted molars) [1, 2, 6]. Radiographically, affected teeth may have crowns with bulbous appearance and accentuated constriction in the cementoenamel junction, narrowed roots, and large root canals due to defective dentin formation, giving the teeth a taurodontic appearance. In addition, pulp chambers and root canals can become partially or totally obliterated over time [7].

OI types I and IV are mild condition forms caused by either COL1A1 or COL1A2 genes mutation [1]. OI type I is characterized by blue sclerae, normal height, and mild short stature. OI type IVB is characterized by moderately short stature, grayish or white sclerae, and dentinogenesis imperfecta [1, 8]. Thus, we believe that our patient was affected by OI type IVB (Table 1).

Although there is no definitive cure for OI, this disease may be treated with bisphosphonate in association with adjuvant orthopedic and physical therapies [4]. Bisphosphonate regiment in children with OI aims to increase bone mineral density and reduce the incidence of osteoporotic fracture [9]. Commonly, the drug of choice is pamidronate (0.5–1.5 mg/kg) which is administered by intravenous perfusion for 3 days at 4-month intervals over a period of 3-4 years [6]. However, of the three drugs studied, there was a greater rate of jaw bisphosphonate-related osteonecrosis with the use of pamidronate (74.6% of cases) and zoledronate in adults [8]. Thus, since bisphosphonates show a risk of development of jaw osteonecrosis, dental extractions should be carefully discussed betwenn health care professionals to avoid this serious complication [3, 6]. Bisphosphonate-related osteonecrosis of the jaw is characterized by necrotic bone presented for ≥8 weeks in a patient with history of bisphosphonate therapy [3, 10, 11]. In a recent cohort study of 109 patients with bisphosphonate-related osteonecrosis of the jaw, the etiology of this condition in 51.4% of the cases were surgical interventions such as dental extraction, implant placement or periodontal surgery [10].

At present, there is no established clinical protocol for children with OI undergoing eruption of permanent dentition, needing deciduous tooth extraction and orthodontic/orthopedic therapy [6, 12]. In these situations, some authors have recommended bisphosphonate therapy discontinuation 4 months prior to dental surgery in interventions involving osteotomy, while 8–15 days discontinuation would suffice for simple dental extractions. In both cases, antibacterial prophylaxis is necessary [3, 6]. It must also be noted that although bisphosphonate therapy withdrawal may not interfere with the bisphosphonate previously incorporated into the bone, it could facilitate the healing process of the injured tissues by preventing the antiangiogenic effect of bisphosphonates [13]. In the present case, surgical procedures were carried out with bisphosphonate therapy maintenance and without antibacterial prophylaxis, since no risk of infection was observed in the transoperative period. Ideally, bisphosphonate therapy should only be initiated after dental treatment is finalized; however, in most clinical situations, drug therapy has been previously instituted. In these circumstances, the American Association of Oral and Maxillofacial Surgeons (AAOMS) [3] and the Japanese “Allied Task Force Committee of Bisphosphonate-Related Osteonecrosis of the Jaw” [11] suggest that dental procedures should be performed before bisphosphonate dose reaches a high level. They also recommend nonsurgical conservative dental treatments and the establishment of preventive measures providing adequate oral hygiene to reduce the risk of bone necrosis and obtaining informed consent from the patient/legal guardian before dental interventions. Presently, a surgical intervention was adopted to (1) improve oral hygiene, (2) prevent further discomfort, and (3) allow proper dental eruption. In addition, this decision was based on the child's mother request. Although the evidence for bisphosphonate-related bone necrosis in children is considered scarce, the risk of this condition should be discussed when invasive dental procedures are needed.

In summary, OI requires a multidisciplinary approach, highlighting the participation of pediatric dentists in its early recognition through oral and radiographic manifestations such as DI. Children with OI, scheduled to receive intravenous bisphosphonate therapy, should be appropriately evaluated by a pediatric dentist to provide the rational for an adequate treatment planning and to identify any risk factors for bisphosphonate-related osteonecrosis. Even though this condition is considered rare among children, osteonecrosis may be a life-threatening event in young patients.

## Figures and Tables

**Table 1:** Classification of osteogenesis imperfecta into the four most frequent types^∗^.

Types∗∗	Main features
Type IA	Mild absence of bone deformity Caused by mutations in COL1A1 or COL1A2 Affects approximately 60–70% of carriers of OI Blue sclerae Normal stature

Type IB	Dentinogenesis imperfecta associated with type IA

Type II	More severe form High antenatal mortality rate due to extreme bone fragility

Type IIIA	Presence of progressive bone deformities and short stature Presence of extreme ligamentous laxity Affects approximately 20% of carriers of OI

Type IIIB	Dentinogenesis imperfecta associated with type IIIA

Type IVA	Mild deformities with variable degrees of short stature Diversified form Blue and white-grayish sclerae Associated hearing loss Mutations in COL1A1 or COL1A2 Affects approximately 10% of carriers of OI

Type IVB	Dentinogenesis imperfecta associated with type IVA

∗Modified from Sillence et al. [14].

∗∗Additional subtypes: I-A and I-B (Levin et al. [15]); III-A and III-B (adopted in the present study); IV-A and IV-B (Paterson et al. [16]).

*Footnote*. OI may also be classified into types V (Glorieux et al. [17]), VI (Glorieux et al. [18]), VII (Ward et al. [19]), and VIII (Cabral et al. [20]) based on clinical and bone histological parameters.
